# Prevention of Loss of Muscle Mass and Function in Older Adults during COVID-19 Lockdown: Potential Role of Dietary Essential Amino Acids

**DOI:** 10.3390/ijerph19138090

**Published:** 2022-07-01

**Authors:** Sanghee Park, Yewon Chang, Robert R. Wolfe, Il-Young Kim

**Affiliations:** 1 Department of Molecular Medicine, College of Medicine, Gachon University, Incheon 21999, Korea; sangheepark1@gmail.com; 2 Korea Mouse Metabolic Phenotyping Center, Lee Gil Ya Cancer and Diabetes Institute, Gachon University, Incheon 21999, Korea; 3 Department of Health Sciences and Technology, Gachon Advanced Institute for Health Sciences and Technology, Gachon University, Incheon 21999, Korea; jjangye1@gachon.ac.kr; 4 Center for Translational Research in Aging and Longevity, Department of Geriatrics, Donald W. Reynolds Institute on Aging, University of Arkansas for Medical Sciences, Little Rock, AR 72205, USA; rwolfe2@uams.edu

**Keywords:** COVID-19, physical inactivity, sarcopenia, essential amino acids, muscle mass, muscle quality, protein synthesis, EAA availability

## Abstract

As the COVID-19 pandemic became a global emergency, social distancing, quarantine, and limitations in outdoor activities have resulted in an environment of enforced physical inactivity (EPI). A prolonged period of EPI in older individuals accelerates the deterioration of skeletal muscle health, including loss of muscle mass and function, commonly referred to as sarcopenia. Sarcopenia is associated with an increased likelihood of the progression of diabetes, obesity, and/or depression. Well-known approaches to mitigate the symptoms of sarcopenia include participation in resistance exercise training and/or intake of balanced essential amino acids (EAAs) and high-quality (i.e., containing high EEAs) protein. As the pandemic situation discourages physical exercise, nutritional approaches, especially dietary EAA intake, could be a good alternative for counteracting against EPI-promoted loss of muscle mass and function. Therefore, in the present review, we cover (1) the impact of EPI-induced muscle loss and function on health, (2) the therapeutic potential of dietary EAAs for muscle health (e.g., muscle mass and function) in the EPI condition in comparison with protein sources, and finally (3) practical guidelines of dietary EAA intake for optimal anabolic response in EPI.

## 1. Introduction

As the coronavirus SARS-CoV-2 (coronavirus disease 2019, COVID-19) outbreak has spread around the world, extended periods of time restricted to home have become routine. The associated enforced physical inactivity is significant, which inevitably leads to muscle atrophy [1]. Despite a global endeavor to end the pandemic, the physiological effects of the COVID-19-associated EPI may persist. Even potentially more problematic, as mandated EPIs end older individuals may never be entirely reversed. Further, older individuals who have experienced EPI may never resume a high level of activity. Prolonged EPI negatively affects other clinical conditions such as obesity, insulin resistance, diabetes, cardiovascular disease, and cancer. Therefore, there is a growing and urgent need to discover therapeutic means to ameliorate the deterioration of muscle health induced by EPI in the COVID-19 era.

Despite significant research endeavors, no effective therapeutics to prevent loss of muscle mass in aging has been discovered. Resistance exercise and consumption of increased dietary protein and/or essential amino acids (EAAs) are the two most potent and safe anabolic stimuli to counteract the loss of muscle mass and strength in sarcopenia. However, given the pandemic situation of staying at home or in quarantine, it is likely to be difficult for older adults to perform sufficient exercise to reverse the adverse effects of EPI, particularly with concurrent clinical conditions. The most practical and effective means of slowing the loss of muscle mass and function in EPI is by optimal nutrition, particularly including adequate intake of free dietary EAAs, which can be formulated with the optimal composition [2,3]. An appropriate composition of dietary EAAs induces a greater anabolic response (i.e., an increase in net protein synthesis) than isocaloric protein intake [4]. The superior efficacy of EAAs in inducing an anabolic response translates to improved physical performance in older adults [5,6,7].

In this review, we discuss the potential role and underlying mechanisms of dietary EAAs in counteracting the deterioration of muscle mass and function in older adults in the EPI environment.

## 2. COVID-19-Induced Physical Inactivity and Its Influence on Sarcopenia

Physical inactivity, defined as not meeting the minimum of physical activity to preserve physical fitness and health as recommended by health organizations such as the American College of Sports Medicine (ACSM), American Heart Association (AHA), and the World Health Organization (WHO), is considered to be a leading risk factor for chronic diseases such as obesity, type 2 diabetes, or dyslipidemia [8]. Loss of skeletal muscle mass and function may become severe as the amount of physical activity is reduced [9,10]. Although several social and environmental factors (e.g., sedentary lifestyle) can be overcome by motivating people to participate in exercise programs, there are various conditions that limit the ability of individuals to exercise, such as fracture, hospitalization, and pandemic situations such as COVID-19. In this context, preventative measures associated with the COVID-19 pandemic have threatened muscle health, particularly in older adults, leading to acceleration of the progression to sarcopenia [11,12,13].

Individual isolation has become common due to the global impact of COVID-19. Governments have recommended social distancing to prevent further infection from spreading via social gatherings and any group activities, which in turn leads to the inevitable reduction of outdoor activities as well as workouts in a gym. Pinto and her colleagues reported that COVID-19 promotes the global decline of habitual physical activity, which contributes to the progression of chronic diseases such as obesity, sarcopenia, and diabetes [14]. People have also restricted their physical activity as they are required to quarantine when traveling abroad or having contact with people infected by COVID-19. According to a report by Fitbit Co. (Fitbit; San Francisco, CA, USA), an American consumer electronics and fitness company, step count data measured by their wearable devices were significantly decreased around the world due largely to the impact of COVID-19 [15]. Reduction of step numbers induced loss of muscle mass via accelerating impairments of muscle protein synthesis (MPS) in fasted and fed conditions [16] and vascular dysfunction (i.e., vasodilation and thus delivery of nutrients) within 2 weeks in older adults [9]. In addition, changes in habitual eating patterns toward increased carbohydrate intake during the COVID-19 era [17] can further exacerbate the EPI-induced loss of muscle mass.

Physical inactivity can become a life-threatening problem in older adults with clinical conditions [18]. For example, the rate of muscle mass reduction is most extensive in the first two weeks of confinement of older individuals in the intensive care unit (ICU) for treatment of COVID-19 [9]. Further, reduction of physical activity is closely associated with suppressed food consumption, thereby worsening muscle health. Previous studies have shown that in case of hospitalization, 21% of aged patients only consume 50% of their dietary requirement when hospitalized [19], implying that rapid deterioration of muscle health in hospitalization was due to the combined effect of physical inactivity and energy deficit. Moreover, in the recovery from diseases, older patients suffer from the difficulty of returning to their daily routines and, in turn, suffer delayed rehabilitation [20] from poor muscle mass and strength. These results suggest an urgent need for finding an effective means to reduce muscle loss resulting from inactivity.

**Figure 1:**
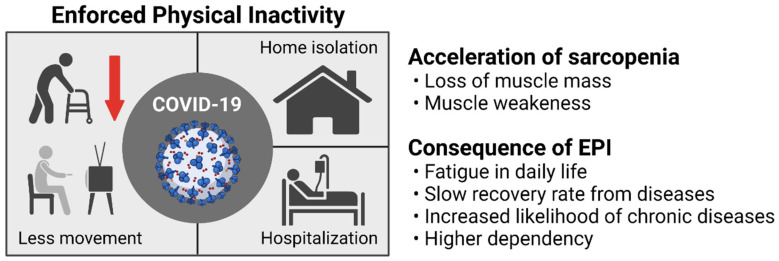
The COVID-19 pandemic spread around the world, forcing people, particularly older adults, to physically restricted conditions, including home isolation and hospitalization. These conditions resulted in further reductions in daily physical activity, leading to deterioration of muscle health (loss of muscle mass and function) in older adults, which is closely associated with worse consequences, including fatigue in daily life, slow recovery rate from diseases, increased likelihood of chronic disease, and thus inability of their independent life. The figure was created by BioRender.

Decreased muscle mass in older adults is associated with increased mortality and reduced quality of life [15]. Muscle cross-sectional area and fiber numbers as well as rates of MPS are all decreased with aging [2,21], which in turn increases the risk of falling or fracture and impairs recovery from various diseases [22,23]. Given their often-fragile physiological conditions, fear of contracting COVID-19, and a higher likelihood of hospitalization, older adults are more susceptible to the adverse effects of EPI, as described in Figure 1. As a result, there is a reason for serious concern about the effects of procedures designed to mitigate the spread of COVID-19 that contribute to EPI in older individuals.

**Figure 2:**
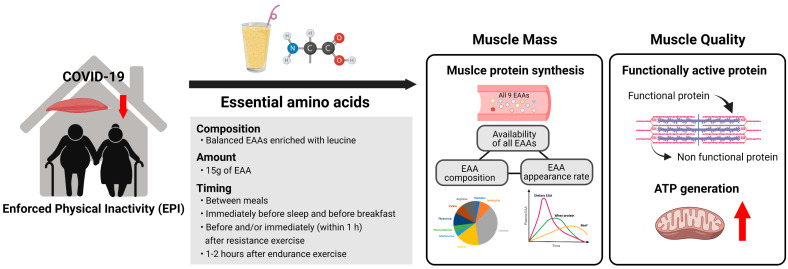
Summary of beneficial impacts of dietary EAAs on aging muscle in EPI. To maximize the benefits of dietary EAAs, three key factors need to be optimized: (1) composition (i.e., balanced EAAs), (2) amount of EAA intake (~15 g EAAs per intake), and (3) timing of EAA intake (e.g., between meals, before and/or post-exercise). Optimal EAA intake leads to greater stimulation of muscle protein synthesis (MPS) through increased availability of plasma EAAs and, in turn, gains in muscle mass and function as well as muscle quality (force production for a given muscle mass). Improvement of muscle quality is likely driven by enhancements in muscle protein turnover (i.e., synthesis and breakdown), replacing old, non-functional proteins with new, functional proteins and mitochondrial function (providing energy for the protein turnover and for muscle contractile activity). The figure was created by BioRender.

For the reasons discussed above, there is a growing need for an effective countermeasure to EPI to prevent older people from further loss of muscle mass and strength [1,24]. Although resistance exercise is an effective means, nutritional approaches (i.e., dietary protein from natural food sources, isolated proteins such as whey protein, and free EAA supplements) are more practical for older adults. Optimally formulated free EAA compositions may be most effective in alleviating the EPI-mediated decline in muscle health without adverse side effects. Thus, we discuss in the following sections how dietary EAA intake can improve or at least preserve muscle mass and physical performance in aging muscle in EPI (Figure 2).

## 3. Superiority of Dietary EAAs for Muscle Growth

In general, muscle growth occurs when the rate of protein synthesis is greater than the rate of protein breakdown (i.e., positive protein balance), whereas muscle loss occurs when the rate of breakdown exceeds the rate of synthesis [25]. Although MPS is only part of the basis for changes in muscle mass, a change in MPS, but not muscle protein breakdown (MPB), is the primary basis for muscle growth in the postprandial state, which in turn can lead to gains in muscle strength [26,27]. Here, we discuss the following factors whereby EAAs can affect MPS: (1) availability of all of the EAAs, (2) composition or profile of EAAs, (3) the appearance rate in the circulation of absorbed EAAs, and (4) stimulation of signaling responsible for MPS. Then, we cover the role of dietary EAAs for changes in MPS in a variety of circumstances, including prolonged physical inactivity and metabolic dysfunction.

### 3.1. All EAAs Work as a Team for Making New Proteins

Synthesis of new proteins typically requires all 20 amino acids, which consist of 9 EAAs and 11 non-essential amino acids (NEAAs). Unlike NEAAs, EAAs need to be provided exogenously, typically in the context of a food source, since our body cannot produce EAAs. Over the past few decades, clinical studies have shown that EAAs, but not NEAAs, simulate MPS [28,29]. EAAs induce a greater increase in MPS than isonitrogenous high-quality protein [30]. The greater stimulatory effect of EAAs is due in part to their greater and more rapid availability in plasma following intake, as compared to an intact protein, due to the more rapid and complete absorption of EAAs in the free form [3,31,32,33]. In addition, the amount of EAAs contained in a composition of only free EAAs is greater on a gram/gram basis than any intact dietary protein, leading to a greater increase in MPS [4,30]. Consumption of an EAA composition also stimulates the utilization of endogenous NEAAs, as indicated by the suppression of plasma NEAA concentrations [3], which may reduce the metabolic burden on the liver and kidneys. A shortage of any single or more of EAAs can lead to an impairment of MPS stimulation. For example, Louard et al. demonstrated an absence of stimulation of MPS during an intravenous infusion of BCAA (leucine, isoleucine, and valine), accompanied by reductions in other EAA concentrations in the plasma [34]. Similarly, leucine supplementation (7.5 g per day for 3 months) showed no change in muscle growth in healthy older adults [35]. These results emphasize the importance of the plasma availability of all EAAs for stimulation of MPS.

### 3.2. Role of EAAs in Stimulation of MPS

Muscle is composed of specific ratios of 20 AAs, indicating that a particular amount of each EAA is required to produce new muscle protein. It was therefore thought that mimicking the EAA profile in muscle protein would be the most effective composition to stimulate muscle protein synthesis. However, clinical research discovered that the optimal composition of EAAs is different from the EAA profile of muscle protein for several reasons [5,31,36,37]. First, leucine is oxidized in muscle in addition to being incorporated into muscle protein by MPS. Second, lysine transmembrane transport from the blood into muscle is slow, and thus lysine availability in muscle does not increase in proportion to its prevalence in the composition of EAAs. Therefore, the optimal formulation of an EAA composition to stimulate MPS will have disproportionately greater amounts of leucine and lysine as compared to their respective contributions to the composition of muscle protein. In line with this notion, it was shown that a specially formulated composition of all the EAAs containing approximately 35% leucine and 15% lysine (which exceeds their contributions to the composition of muscle protein) effectively overcame anabolic resistance and stimulated MPS [9,20] and improved functional performance in older adults [5].

### 3.3. Role of Plasma EAA Appearance Rate on Stimulation of MPS

The stimulation of MPS depends largely on the rate of EAA appearance in the circulation following intake of dietary EAA sources such as mixed meals, protein food sources, or EAA supplements [30,38]. Contrary to protein food sources, dietary EAAs do not require digestion before appearing in the circulation, thus inducing rapid and complete appearance rates of EAAs in the circulation. Previous studies have shown that the rate of plasma EAA appearance varies depending on amino acid sources [39,40,41], and the speed of the digestion significantly influences the magnitude of MPS [42]. For example, free EAAs are rapidly absorbed, which leads to higher peak concentrations of plasma EAAs and greater stimulation of MPS compared to casein, whey, or ground beef [38,39,43]. As EAAs are transported against concentration gradients into muscle cells (i.e., ATP-dependent active transport), the elevations of plasma EAA concentrations accelerate inward uptake of EAAs into muscle, leading to greater stimulation of MPS. The importance of the speed of increase in plasma EAA concentrations is further confirmed by a study in which consumption of 12.6 g of AA supplement containing high EAAs induced a faster and greater appearance of plasma EAAs compared to whey protein, resulting in a greater improvement of MPS [30]. These data indicate that a rapid appearance rate of EAAs following a balanced dietary EAA intake is a key to enhance MPS in older adults in EPI.

### 3.4. Role of EAAs on Stimulation of MPS Signaling

Given sufficient availability of all the EAAs, dietary leucine can activate intracellular signaling responsible for the stimulation of MPS, i.e., the mTOR signaling pathway. Leucine may also act as an insulin secretagogue, leading to further stimulation of MPS through the insulin receptor [44]. Studies have shown that MPS signaling machinery, including mTOR, p70 S6 kinase, and initiation factor 4E-binding protein 1, can be activated by leucine, insulin, or both [44,45]. Leucine can positively influence MPS, independent of activating insulin or mammalian target of rapamycin (mTOR) signaling pathway. Anthony et al. demonstrated that leucine administration increases the anabolic response in littermates of diabetic rats without changes in serum insulin or activation of mTOR signaling [46]. While insulin is known as a potent stimulator of MPS, insulin typically has a greater influence on the suppression of MPB than stimulation of MPS [47]. The underlying mechanism by which insulin suppresses MPB is not clear but possibly involves suppression of the ubiquitin-proteasome system, including atrogin-1 and MuRF-1 and autophagy-lysosomal degradation [48,49]. Whereas leucine alone may increase the signaling transduction and insulin secretion, a surfeit of all the EAAs is necessary for these actions of leucine to enhance the anabolic response.

### 3.5. Clinical Evidence: Dietary EAAs Are More Effective in Inducing MPS Than High-Quality Protein

It has been repeatedly demonstrated in clinical studies that dietary EAAs are effective in enhancing MPS and thus lean body mass (reflecting muscle mass) in older adults in various circumstances. For example, a small amount of dietary EAA intake (7.5 g of EAA, twice a day) for 12 weeks significantly increased lean body mass in healthy older women [27]. In addition, dietary EAA intake as small as 3 g stimulated MPS to a similar extent as 20 g of whey protein in older adults [50]. These data demonstrated that a relatively small amount of dietary EAA intake efficiently increases MPS in older adults. Free EAAs may also prevent loss of muscle mass in EPI. Reduced physical activity (reflected by the number of steps taken in a day) or enforced bed rest reduces MPS in older adults [10,16]. However, free EAA intake (15 g per meal) throughout 10 days of enforced bed rest, which mimics severe physical inactivity conditions such as being in an intensive care unit, prevented loss of muscle mass in older adults [10]. Thus, dietary EAAs can be widely applicable as a countermeasure in EPI conditions, particularly in hospitalized older patients vulnerable to accelerated loss of muscle mass due to EPI.

Metabolic dysfunction in aging can be ameliorated by free EAAs, likely through improvements in muscle mass. Eleven g of EAA + arginine supplementation twice a day for 16 weeks in older adults with insulin resistance increases lean body mass [6]. Further, leucine-enriched EAAs produce a greater MPS in older adults with impaired glucose tolerance [6] and with anabolic resistance [2]. In addition, a balanced EAA-enriched beverage induces approximately five times greater protein accretion than a whey protein beverage for a given caloric content in older adults with heart failure [3].

Taken together, these data indicate that the optimal stimulation of MPS and thus better preservation of muscle mass in the EPI situation can be best accomplished by the provision of a sufficient amount of a balanced composition containing all the EAAs in free form.

## 4. Roles of EAAs in Improving Muscle Quality

Dietary EAA intake, particularly when combined with resistance exercise training, increases muscle mass, strength, and function as well as muscle quality (defined as muscle strength or function per unit of muscle mass) [5,6,27]. Older adults typically experience a faster loss of muscle strength than muscle mass (i.e., loss of muscle quality). However, it seems that EAA intake can counteract the loss of muscle quality in older adults. For example, recent clinical studies reported that consumption of free EAA as a dietary supplement (from 8 g to 22 g/day) for 8–24 weeks enhanced hand grip strength, leg strength, or walking speed without gains in lean body mass in older adults, indicating improved muscle quality [5,7,26]. The improvement of muscle quality may stem in part from increased muscle protein turnover, leading to replacements of old, non-functional with new, functional contractile [6,51,52,53] and mitochondrial [52,54] proteins.

### 4.1. Functional Contractile Proteins in Skeletal Muscle

The decline of strength and function with advanced age is only partially explained by loss of muscle mass. This observation implies a loss of muscle quality, which is likely due in part to a progressive accumulation of non-functional proteins in the muscle through suppressed muscle protein turnover [6,36]. Stimulation of MPS by supplementation of the diet with free EAAs was accompanied by improvement of muscle strength in older adults [6,52] and occurred independently of changes in muscle mass, indicating an improvement in muscle quality [5]. Consistent with this observation, we have recently shown that free EAA intake improves maximal load-carrying capacity against gravity, tested on a ladder as well as MPS in mice without changes in muscle mass [51], supporting the idea that newly synthesized proteins are functionally intact as compared to pre-existing protein [52].

### 4.2. Mitochondrial Biogenesis

Mitochondrial function declines progressively with aging [55]. Recent studies demonstrated that dietary EAA supplementation improves walking distance for 6 min in older adults [5,6], potentially due in part to EAA-mediated stimulation of mitochondrial biogenesis [56]. The possible role of mitochondrial biogenesis is supported by the improved physical function in the absence of increased lean body mass or muscle mass in older adults [5,6] and rodents [51]. Increased EAA intake can stimulate the synthesis of oxidative enzymes in mitochondria and promote ATP generation [51,56]. It is likely that dietary EAAs enhance the capacity of TCA cycle intermediates in mitochondria to replenish energy depletion. Marquis et al. have shown that EAA intake in older adults increases TCA intermediates [48]. In agreement with these findings, Scarabelli et al. have also shown that EAA supplementation enhances ATP content in muscle and the rate of ATP replenishment in gastrocnemius muscle following exhaustive exercise in old rats [54]. Furthermore, dietary EAA intake significantly increased muscle quality, which is closely associated with mitochondrial protein synthesis rate [54].

To summarize, supplemental EEA consumption can improve muscle quality via (1) increased functionally active protein, (2) mitochondrial biogenesis, and (3) efficacy in energy-generating machinery in mitochondria. All of these processes are likely to be mediated by all stimulation of protein turnover.

## 5. Practical Guidelines for Consumption of Free EAA Supplemental Intake for Older Adults

EAAs can serve as a promising nutraceutical to attenuate the accelerated progression of sarcopenia in the COVID-19 era. Here, we provide conceptual guidelines for dietary EAA intake to maximize muscle health in terms of dosage, composition, timing, and frequency of dietary EAA intake.

### 5.1. Dosage

The first key to optimal EAA supplementation is determining the preferred dosage. The optimal amount of EAAs per intake for boosting MPS in older adults is approximately 15 g, as it was shown that the stimulation of MPS is linearly increased from a dose of 3 g up to 15 g of dietary EAA intake, which stimulates MPS to the same extent as 40 g of whey protein [57]. However, if a dosage of 15 g of EAAs is not practical for some reason, a very small amount of EAAs can be remarkably effective. Bukhari et al. demonstrated that 3 g of leucine-enriched EAAs induces a similar anabolic response of muscle that is induced by intake of 20 g of whey protein in older women at rest [50]. This superior efficiency of dietary EAAs in the enhancement of MPS provides additional advantages: reduced nitrogenous waste product and less calorie intake. First, consumption of dietary protein increases plasma NEAAs, thereby increasing the production of nitrogenous waste products (ammonia and urea) in the liver, which must be cleared by the kidneys. This, in turn, may give a burden to those who suffer from liver disease or renal failure. On the contrary, consumption of dietary EAAs actually reduces plasma NEAA concentrations by incorporating NEAAs with exogenously introduced EAAs to make new proteins [3], possibly relieving the burden on the liver and kidney while maintaining muscle health. Second, consumption of dietary protein rather than an appropriate free EAA composition provides additional calorie intake to achieve a similar stimulation of MPS. For example, in order to achieve maximal MPS from different protein sources, approximately 40 g of whey protein or 200 g of ground beef is required, while 15 g of EAA intake is sufficient. The caloric intake of 15 g of EAA is 60 kcal (assuming caloric-free flavorings), as compared to a minimum of 160 kcal and 350 kcal for whey protein and beef, respectively.

### 5.2. Composition

The second key to dietary EAAs in preserving muscle mass and function is to consume an optimal composition of all nine EAAs (i.e., balanced EAAs). The maximal synthesis of new muscle protein requires the provision of all the EAAs, and a specific proportion of individual EAAs determined by clinical studies. For better results, it is recommended to enrich leucine in the balanced EAA composition for older adults to maximize net gain in muscle protein via MPS stimulation [20]. Katsanos et al. found that older adults, unlike young subjects, had limited responsiveness of MPS to a composition of dietary EAAs containing 26% of leucine, which is overcome by increasing the leucine content to 41% [31]. Additionally, although not an EAA, specific NEAAs such as arginine and citrulline can augment the MPS response to EAA supplementation [41] through various mechanisms, including improvement in muscle blood flow due to stimulation of nitric oxide production [51]. For example, arginine is the direct precursor of nitric oxide, a potent vasodilator, and its bioavailability in the blood is more effectively increased when citrulline is consumed compared to isonitrogenous arginine due to extensive consumption of arginine by the liver. Thus, it is likely that the fortification of EAAs with citrulline enhances AA delivery to muscle, the main driver for the stimulation of MPS [58,59,60]. Leucine-enriched EAAs with the addition of arginine significantly enhance strength and physical function measured by one repetition maximum and five-step test in older adults, respectively [6]. Thus, a balanced EAA composition enriched with leucine and either arginine of citrulline can maintain muscle mass and function in older adults by overcoming anabolic resistance and increasing nutritional delivery.

### 5.3. Timing and Frequency

The third key to dietary EAAs in preserving muscle mass is the optimal timing of consumption. EAA intake between meals will help to preserve the postprandial rate of MPS in conditions in which age-related anorexia and weakened masticatory activities in older adults [61,62] induce limitations to achieving a sufficient EAA consumption from traditional protein food sources. A previous study demonstrated that supplement intake following a meal significantly increased plasma EAA concentration, similar to the response to a large protein intake in a meal [63], suggesting that EAA consumption at 2–3 h following a meal can sustain plasma EAA availability and induce optimal MPS in older adults. EAA intake just before bedtime or before breakfast (where protein breakdown dominates over protein synthesis) can prevent sustained muscle breakdown and provide a favorable environment for the enhancement of MPS [64,65,66]. A dose of EAA supplement before sleep sustains plasma EAA concentrations and increases whole-body net protein balance [64] and MPS [65,66] during sleep. Third, if possible, it is recommended to take the balanced EAA supplement in conjunction with resistance exercise [67,68]. In this case, the consumption of balanced EAAs immediately before [69] or within 1 h [32,69] after the completion of resistance exercise or during the exercise [70] will lead to greater stimulation of MPS. As resistance exercise stimulates protein breakdown during the exercise session, balanced EAA intake before or during resistance exercise not only suppresses protein breakdown but also stimulates protein synthesis. In addition, following a bout of exercise, EAA intake augments MPS due to the increased sensitivity of MPS to plasma EAA appearance. It was demonstrated that EAA intake immediately before resistance exercise largely amplifies MPS [32]. Studies also showed that supplement ingestion at 15 min following initiation of resistance exercise or 1 h post-exercise stimulates whole-body net balance and MPS in older adults to a rate not different from younger counterparts [60,61]. Fourth, although not directly affecting changes in muscle mass, endurance exercise is worth mentioning, as it can improve metabolic and cardiovascular health. When performing endurance exercise, it would be beneficial to take dietary EAAs 1 h after endurance exercise. While consumption of EAAs before endurance exercise may also be beneficial, the effectiveness of EAAs may be diminished by increased oxidation of leucine during exercise [71].

It is recommended for older adults to consume up to 15 g of balanced dietary EAAs before breakfast, between meals, and immediately before bedtime to maximize the anabolic response (i.e., gains in muscle) [72]. In addition, if possible, EAA intake within 1 h before or after resistance exercise or during the exercise can augment muscle anabolic response. If this schedule is too aggressive for an individual, the benefit will be obtained from smaller doses of EAAs.

## 6. Conclusions

Maintaining muscle mass and function with aging has become increasingly challenging for older individuals in the current circumstance of COVID-19 owing to the EPI-mediated acceleration of loss of muscle mass and function, particularly in older individuals. COVID-19-based EPI induces loss of muscle mass and function similar to other catabolic circumstances, including restriction to an intensive care unit or serious trauma. While there exist no safe and effective drugs to reverse sarcopenia, dietary EAAs are a promising nutraceutical to effectively prevent loss of muscle mass and quality in EPI or in various disease conditions such as cancer cachexia, heart failure, and chronic kidney disease. It is important to consume balanced EAAs with optimal dosage, composition, timing, and frequency in order to achieve superior effects as compared to other potentially anabolic nutrients, including high-quality protein. EAAs can better preserve muscle mass and also improve muscle quality than any intact dietary protein. Further, supplementation of the diet with an appropriate composition of free EAAs, when combined with exercise, could enhance gains in muscle mass and physical performance in older adults, with associated health and social benefits. Due to the fact that previous studies were largely conducted in normal conditions, more clinical evidence with respect to the effect of EAA supplementation needs to be accumulated in the condition of EPI.
